# Comparing *in vivo* bioluminescence imaging and the Multi-Cruzi immunoassay platform to develop improved Chagas disease diagnostic procedures and biomarkers for monitoring parasitological cure

**DOI:** 10.1371/journal.pntd.0010827

**Published:** 2022-10-03

**Authors:** Amanda Fortes Francisco, Ursula Saade, Shiromani Jayawardhana, Hans Pottel, Ivan Scandale, Eric Chatelain, Peter Liehl, John M. Kelly, Maan Zrein

**Affiliations:** 1 Department of Infection Biology, London School of Hygiene and Tropical Medicine, Keppel Street, London, United Kingdom; 2 Infynity Biomarkers, Lyon, France; 3 Department of Public Health and Primary Care, KU Leuven Campus Kulak Kortrijk, Kortrijk, Belgium; 4 Drugs for Neglected Diseases initiative (DNDi), Geneva, Switzerland; University of Texas at El Paso, UNITED STATES

## Abstract

**Background:**

Chagas disease is caused by the protozoan parasite *Trypanosoma cruzi* and is a serious public health problem throughout Latin America. With 6 million people infected, there is a major international effort to develop new drugs. In the chronic phase of the disease, the parasite burden is extremely low, infections are highly focal at a tissue/organ level, and bloodstream parasites are only intermittently detectable. As a result, clinical trials are constrained by difficulties associated with determining parasitological cure. Even highly sensitive PCR methodologies can be unreliable, with a tendency to produce “false-cure” readouts. Improved diagnostic techniques and biomarkers for cure are therefore an important medical need.

**Methodology/Principal findings:**

Using an experimental mouse model, we have combined a multiplex assay system and highly sensitive bioluminescence imaging to evaluate serological procedures for diagnosis of *T*. *cruzi* infections and confirmation of parasitological cure. We identified a set of three antigens that in the context of the multiplex serology system, provide a rapid, reactive and highly accurate read-out of both acute and chronic *T*. *cruzi* infection. In addition, we describe specific antibody responses where down-regulation can be correlated with benznidazole-mediated parasite reduction and others where upregulation is associated with persistent infection. One specific antibody (IBAG39) highly correlated with the bioluminescence flux and represents a promising therapy monitoring biomarker in mice.

**Conclusions/Significance:**

Robust, high-throughput methodologies for monitoring the efficacy of anti-*T*. *cruzi* drug treatment are urgently required. Using our experimental systems, we have identified markers of infection or parasite reduction that merit assessing in a clinical setting for the longitudinal monitoring of drug-treated patients.

## Author summary

Infections with the single-cell parasite *Trypanosoma cruzi* are life-long and cause Chagas disease. This can give rise to serious cardiac and/or digestive tract pathology. The current drugs are often non-curative, and patients frequently suffer severe side-effects. There is an international effort to develop improved therapeutic approaches. However, because *T*. *cruzi* persists at very low numbers in discrete tissue/organ foci, it is extremely difficult to unambiguously demonstrate parasitological cure. Even highly sensitive PCR methodologies require long-term follow-up to provide a curative diagnosis that is likely to be reliable. This is problematic for clinical trials and will complicate the roll-out of new drugs at a population level. Here, using experimental mouse models, we have exploited a combination of highly sensitive bioluminescence imaging and an antibody multiplex assay system to identify serological markers of infection and successful treatment. These biomarkers can now be adapted to provide a robust system for the diagnosis of Chagas disease and for confirming the parasitological cure of chronic infections.

## Introduction

Chagas disease results from infection with the insect-transmitted, obligate intracellular parasite *Trypanosoma cruzi*, and is a major public health concern in many areas of Latin America [1, 2]. In humans, the initial acute stage is characterised by widespread distribution of *T*. *cruzi* in tissues and organs, and by a readily detectable parasitemia. The induction of a cellular immune response then results in a major reduction in the parasite load, and by 6–8 weeks post-infection, detection of bloodstream parasites becomes problematic. However, sterile clearance does not occur, and typically the infection transitions to a chronic life-long state [3]. In the acute stage, symptoms are usually mild and transient, although they can occasionally present as myocarditis or meningoencephalitis [4, 5]. 30–40% of infected people eventually develop chronic pathology, with cardiomyopathy and/or digestive megacolon syndromes being the most common outcomes [6, 7]. Chronic symptomatic disease can take decades to become apparent, and the lack of reliable predictive markers to identify those most at risk of disease progression has highlighted the need for new approaches in this area [8, 9].

There is strong evidence that the tissue damage associated with chronic Chagas disease is cumulative and dependent on parasite persistence [10–13]. This provides a rationale to underpin curative drug treatment as a strategy to block or alleviate the development of chronic pathology. The oral nitro-heterocyclic pro-drugs benznidazole and nifurtimox are the only chemotherapeutic treatments available for *T*. *cruzi* infections [14, 15]. Although treatments show efficacy in the chronic indeterminate stage of the disease, long treatment periods (~60 days) and frequent toxic side-effects impact negatively on patient compliance [16–18]. In addition, few of those in the acute or asymptomatic chronic stages are diagnosed, and the percentage of infected individuals offered anti-parasitic treatment, at the time-point when it would be most beneficial, is very small [19]. In response to the urgent need for more effective anti-*T*. *cruzi* therapeutics, there is now a global effort involving the academic, commercial, and not-for-profit sectors [20]. Furthermore, clinical trials aimed at optimising dosing regimens of the currently available drugs are on-going [21].

During the chronic stage, parasites are only intermittently detectable in the bloodstream and infection foci are rare and highly focal at a tissue/organ level [22]. As a result, unequivocal confirmation of parasitological cure is difficult to demonstrate. Even highly sensitive PCR methodologies require long-term follow-up to provide reliable diagnosis [16–17]. Serology-based approaches to assess parasitological cure have also proved to be problematic with seroconversion often requiring many years [23–24]. The identification of robust biomarkers of parasitological cure is therefore crucial to facilitate clinical trials and the subsequent roll-out of new drugs at a community level. The long-term nature and complexity of Chagas disease in humans has meant that predictive experimental models have played a key role in research [25]. One such example is the murine bioluminescence imaging system, based on infections with genetically modified *T*. *cruzi* that express a red-shifted luciferase, which facilitates highly sensitive longitudinal monitoring of parasite burden in a non-invasive manner [26–28]. This has become an important tool for drug testing and for studies on disease pathogenesis. Here, we have combined this pre-clinical model with an antibody multiplex assay system (Multi-Cruzi, InfYnity Biomarkers, Lyon, France) [29–31] as a means of developing improved serological procedures for diagnosis of Chagas disease and for confirming parasitological cure of chronic *T*. *cruzi* infections.

## Methods

### Ethics statement

Experiments were performed under UK Home Office project licenses PPL 70/8207 and P9AEE04E4, with consent of the LSHTM Animal Welfare and Ethical Review Board (AWERB). All protocols and procedures were conducted in accordance with the UK Animals (Scientific Procedures) Act 1986 (ASPA).

### Mice and parasites

Experimental infections were carried out using female BALB/c mice, purchased from Charles River (UK). CB17-SCID mice were bred in-house. They were maintained in individually ventilated cages, under specific pathogen-free conditions, with a 12-hour light/dark cycle, and given food and water *ad libitum*. A *T*. *cruzi* CL Brener line (Discrete Typing Unit (DTU) 6), that constitutively expresses the red-shifted luciferase PpyRE9h [26], was used for infections. BALB/c mice (6–8 weeks old), were injected i.p. with 1x10^3^ bloodstream trypomastigotes obtained from immunodeficient CB17-SCID mice, as outlined previously [27, 28].

### Assessment of drug treatment and bioluminescence monitoring

Chronically infected mice were dosed by oral gavage with benznidazole (Epichem Pty Ltd.) in an aqueous suspension vehicle containing 0.5% (w/v) hydroxypropyl methylcellulose (HPMC), 0.4% (w/v) Tween 80 in Milli-Q H_2_O. For *in vivo* imaging, mice were injected with 150 mg kg^-1^ d-luciferin i.p., and anaesthetized with 2.5% (v/v) gaseous isoflurane in oxygen [27, 28]. They were then placed in IVIS Lumina or Spectrum imaging systems (Caliper Life Science). Images were acquired 10–20 minutes after d-luciferin administration using Living Image 4.7.2. Exposure times were between 1 and 5 minutes, depending on the signal. To estimate parasite burden, whole body regions of interest were drawn using Living Image 4.5.5 to quantify bioluminescence expressed as total flux (photons/second; p/s). The detection threshold was established from uninfected mice.

For *ex vivo* imaging, mice were injected i.p. with 150 mg kg^−1^ d-luciferin and then sacrificed by exsanguination under terminal anesthesia 5 minutes later [32]. They were perfused with 10 ml d-luciferin at 0.3 mg ml^-1^ in DPBS (Dulbecco’s phosphate-buffered saline) via the heart. Organs and tissues were excised, transferred to a dish, soaked in 0.3 mg d-luciferin ml^-1^ in DPBS, and then imaged as for the live mice. At various time points, blood samples (minimum 40 μl) were taken from tail veins into individual sterile tubes containing 10 μl 0.5 M EDTA. They were spun at 1,800 g for 10 minutes (4°C) and the serum fractions collected. Aliquots were stored at -20°C until shipment on dry ice.

### Multiplex Chagas assay

**Fig 1:**
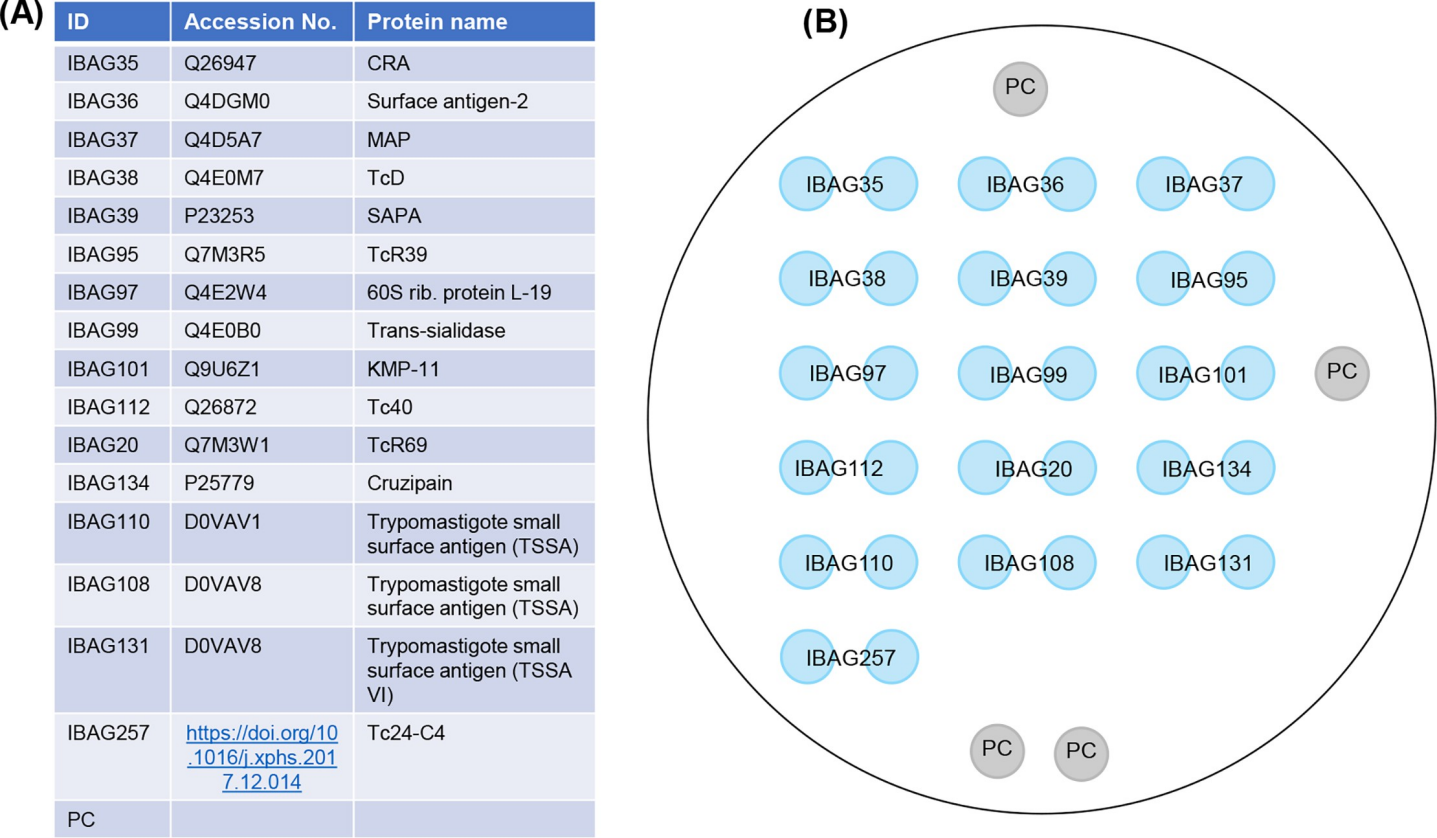
Antigens used in multiplex Chagas assay. (A) List of antigens. (B) Arrangement of antigens spotted in duplicate into the wells of 96-well microtiter plates. Four Positive Control (PC) spots (Methods) were printed to validate the test and give the spatial orientation of the matrix.

The Multi-Cruzi immuno-assay used here is equivalent to the multiplex system described previously [29–31], adapted for mice serum samples. The technology allows multiple antigens to be combined in single wells of a 96-well microplate, using a printing process based on non-contact piezo electric impulsion of a defined volume of an antigenic solution. We used a non-contact volume dispensing system to print 16 *T*. *cruzi* antigens (Fig 1) at the bottom of each well at precise X-Y coordinates, under controlled humidity and temperature. Antigens, selected for proven immunogenic properties, were obtained synthetically and printed in duplicate [29, 30]. In addition, four Positive Control (PC) spots were printed in order to define the spatial orientation and validate the distribution of all the materials (mouse serum samples, conjugate and substrate) in the correct order. The ELISA assay was carried out as previously described [29] using the anti-mouse-IgG secondary antibody conjugated to horseradish peroxidase.

Each plate was read and analysed using a specific microarray reader which acquires high-resolution digital images. An integrated software calculates the pixel intensity for each spot with the background noise subtracted. We calculated the mean value of the duplicated spots to get the net intensity of each antigen. The data were incorporated into a statistical analysis package and analysed accordingly.

### Study design

Three cohorts of 15 BALB/c mice were infected with bioluminescent *T*. *cruzi* (Methods). Cohort size was selected to provide for the expected variability of the immune response, identified in preliminary experiments. When mice had reached the chronic stage (101 days post-infection), they were treated once daily with benznidazole at either 30 or 100 mg kg^-1^ for 5 days, dosing regimens that are generally non-curative or curative, respectively. These curative outcomes were based on mice being bioluminescence negative by both *ex vivo* and *in vivo* imaging following immunosuppressive treatment [33]. In the current experiment, mice were monitored by bioluminescence imaging, with blood samples taken at regular intervals: at days 101, 138, 182, 202, 259 and 300, relative to the day of infection (day 0).

### Statistical analysis

Linear Mixed Model (LMM) analysis was used for the statistical analysis of the longitudinal data, since a mixed effects model has both random and fixed effects. In the current model, treatment group is a fixed effect, while time and mouse are random effects. Longitudinal data are described by the response variable (biomarker intensity), which was repeatedly measured at each group and time. The data prior to start of treatment were disregarded from the LMM analysis. There were no missing value observations. Response variables were the antigen reactivities of 16 biomarkers in the Multi-Cruzi assay and the log-transformed bioluminescence total flux. Independent variables were time (in days after infection) and treatment group (vehicle; benznidazole 30 mg kg^-1^; benznidazole 100 mg kg^-1^). Variance and co-variance were modelled as unstructured (UN). For each subgroup, and for each antigen or bioluminescence variable, slopes and intercepts were calculated and compared. SAS Proc Mixed was used for LMM analysis, from SAS 9.4 (SAS Institute Inc., Cary, NC, USA). The mixed effects model allows the average intercept and slope to be fitted as fixed effects, while taking account of differences between mice and time (random effects).

## Results

### Identification of reactive antigens

Our aim was to use the Multi-Cruzi serology assay to generate immune signatures of disease status before and after benznidazole treatment of *T*. *cruzi-*infected mice (5 days, at either 30 or 100 mg kg^-1^), and to identify biomarkers of chronic disease and curative outcomes. For that purpose, mouse serum samples were screened using the multiplex immuno-assay system containing 16 antigens arrayed in microtiter plate wells as described (Fig 1). The linear mixed model (LMM) analysis results are shown in Table 1.

**Table 1:** Summary of the linear mixed model (LMM) regression output for each biomarker and in each subgroup. Both the intercept and slope of the linear regression of each antigen corresponds to an average over the 15 mice in each group. Intercept > 10. *Blue*: significant p-values (< 0.05); *Red*: high intensity. We have data on several mice where we have treatment group and biomarker reactivity measured at different time points. Where all mice have the same slope and intercept relating reactivity to time and treatment group, a regular multiple linear regression model can be fitted with time and treatment group as the predictor, and reactivity as the response. Biomarker reactivities < 10 were considered below the positive reactivity threshold and were regarded as non-reactive. Slopes compared between treatment groups were assigned as different based on a 5% significance level.

IBAG	BZ 100			BZ 30			Vehicle			Slope difference (p-value)			Interpretation
	Intercept	Slope	p-value	Intercept	Slope	p-value	Intercept	Slope	p-value	BZ100-BZ30	BZ100-Vehicle	BZ30-Vehicle	
**35**	*0*.*7*	*-0*.*00222*	*0*.*8085*	*0*.*8*	*-0*.*0015*	*0*.*87*	*0*.*14*	*0*.*01595*	*0*.*0864*	*0*.*9555*	*0*.*165*	*0*.*1822*	Not reactive
**36**	*6*.*1*	*-0*.*0091*	*0*.*6076*	*4*.*8*	*0*.*002045*	*0*.*908*	*4*.*5*	*0*.*06206*	0.0007	*0*.*6565*	0.0056	0.0186	PersistenceofInfectioninvehicleonly
**37**	*1*.*6*	*-0*.*00568*	*0*.*8268*	*-3*.*9*	*0*.*04108*	*0*.*1165*	*-11*.*9*	*0*.*155*	<0.0001	*0*.*205*	<0.0001	0.0027	PersistenceofInfectioninvehicleonly
**38**	40.6	*-0*.*08696*	0.026	43.1	*0*.*02036*	*0*.*5967*	34.4	*0*.*006806*	*0*.*8595*	*0*.*0512*	*0*.*0876*	*0*.*8031*	TreatmenteffectofBZ100
**39**	97.7	*-0*.*1412*	<0.0001	80.7	*0*.*0358*	*0*.*079*	86.5	*0*.*002115*	*0*.*9167*	<0.0001	<0.0001	*0*.*2404*	TreatmenteffectofBZ100
**95**	*1*.*1*	*-0*.*00347*	0.0023	*1*.*2*	*-0*.*00391*	0.0006	*1*.*2*	*-0*.*00232*	0.0407	*0*.*7817*	*0*.*4727*	*0*.*3199*	Slightly reactive but decreasing
**97**	*0*.*6*	*0*.*0115*	*0*.*1888*	*-0*.*5*	*0*.*005699*	*0*.*5127*	*0*.*43*	*-0*.*00098*	*0*.*9103*	*0*.*6372*	*0*.*312*	*0*.*5873*	Not reactive
**99**	*0*.*2*	*0*.*00061*	*0*.*5695*	*0*.*24*	*-0*.*00047*	*0*.*6601*	*0*.*37*	*-0*.*00086*	*0*.*4232*	*0*.*4759*	*0*.*333*	*0*.*7982*	Not reactive
**101**	*0*.*9*	*-0*.*00102*	*0*.*952*	*-0*.*8*	*0*.*03027*	*0*.*1951*	*-10*.*9*	*0*.*1512*	<0.0001	*0*.*3429*	<0.0001	0.0003	PersistenceofInfectioninvehicleonly
**112**	*5*.*3*	*-0*.*01032*	*0*.*6258*	*10*.*8*	*-0*.*0058*	*0*.*7837*	*10*.*9*	*-0*.*01012*	*0*.*6322*	*0*.*88*	*0*.*9949*	*0*.*8851*	Not reactive
**20**	*0*.*03*	*0*.*00012*	*0*.*362*	*0*.*04*	*0*.*000175*	*0*.*182*	*0*.*04*	*0*.*000133*	*0*.*3111*	*0*.*7641*	*0*.*9428*	*0*.*8194*	Not reactive
**134**	*0*.*06*	*-0*.*00001*	*0*.*9249*	*0*.*05*	*0*.*000024*	*0*.*8518*	*0*.*04*	*0*.*000209*	*0*.*1044*	*0*.*8425*	*0*.*224*	*0*.*3087*	Not reactive
**110**	*0*.*67*	*-0*.*00182*	*0*.*0577*	*0*.*14*	*-0*.*00029*	*0*.*7627*	*0*.*56*	*-0*.*00182*	*0*.*0576*	*0*.*2578*	*0*.*9994*	*0*.*2575*	Not reactive
**108**	*35*.*4*	*-0*.*02184*	*0*.*2991*	*25*.*2*	*-0*.*00808*	*0*.*7*	*35*.*4*	*-0*.*02966*	*0*.*1595*	*0*.*643*	*0*.*7922*	*0*.*4677*	Reactive but no effect
**131**	*19*.*5*	*-0*.*01348*	*0*.*5843*	*20*.*7*	*-0*.*03124*	*0*.*2063*	*26*.*7*	*-0*.*01425*	*0*.*5632*	*0*.*6101*	*0*.*9824*	*0*.*6256*	Reactive but no effect
**257**	112.2	*-0*.*01633*	0.0332	*109*.*5*	*-0*.*00659*	*0*.*3881*	*115*.*6*	*-0*.*01626*	0.034	*0*.*3672*	*0*.*9946*	*0*.*3708*	Highly reactive but regression to the mean?

**Fig 2:**
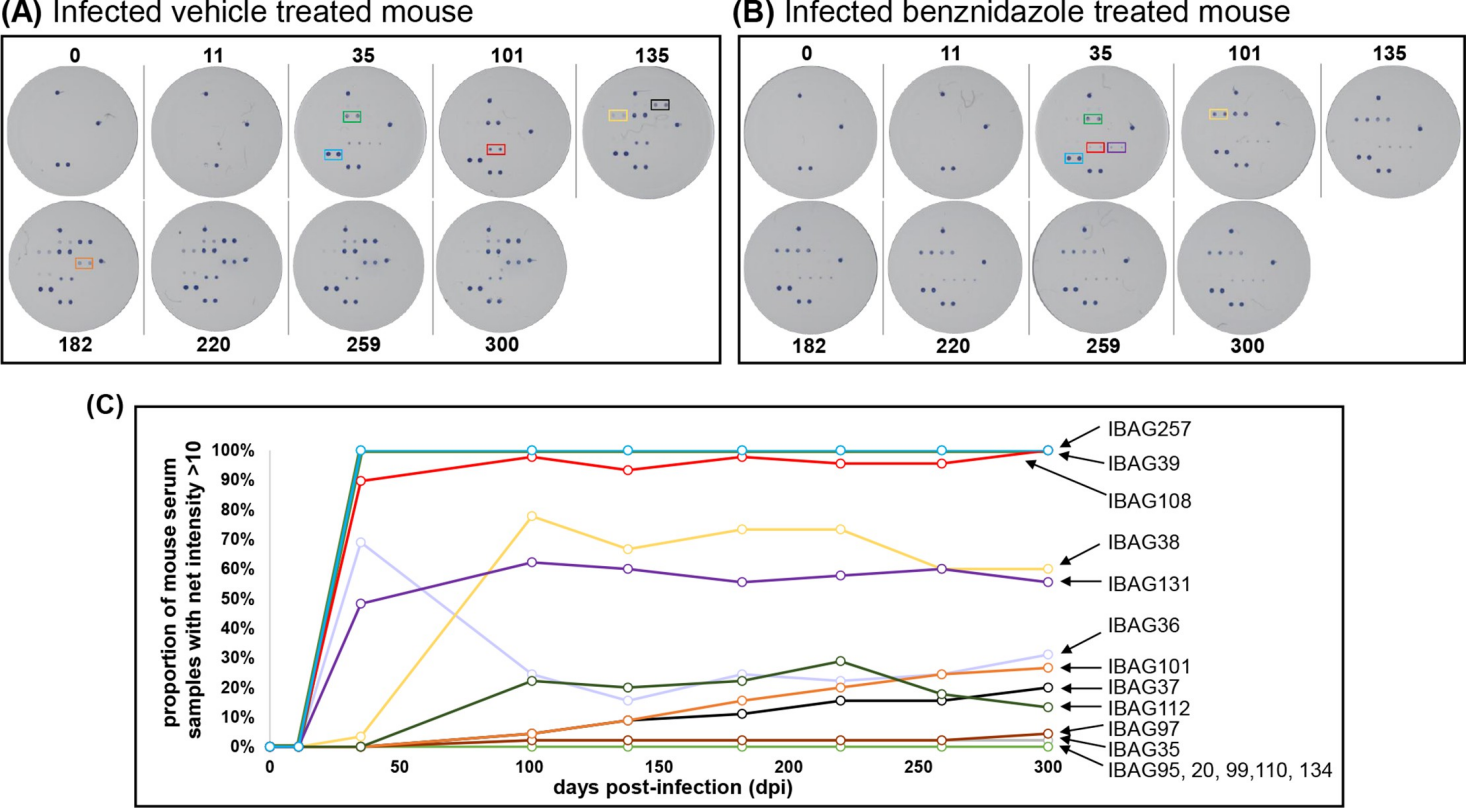
Multiplex immunoassay antigen screening with serum from benznidazole treated and non-treated *T*. *cruzi* infected mice. (A) Images of multiplex assays screened with serum from a vehicle treated infected BALB/c mouse (BN201-4). (B) Images after screening with serum from an infected mouse (BN206-4) that had been subjected to benznidazole treatment (100 mg kg^-1^, 5 days), initiated 101 days post-infection (dpi). The dpi are indicated. Antigens were arrayed in duplicate as in Fig 1. For illustrative purposes, specific antigens are framed as follows: IBAG39 (green), IBAG257 (blue), IBAG108 (red), IBAG37 (black), IBAG38 (yellow), IBAG101 (orange), IBAG131 (purple). (C) Longitudinal data showing the proportion of serum samples reacting with selected antigens at a net intensity >10 (minimum set threshold) at each time point (n = 45). Each IBAG is indicated by an arrow. The curves of the two most reactive antigens IBAG257 (blue) and IBAG39 (green) overlap.

**Fig 3:**
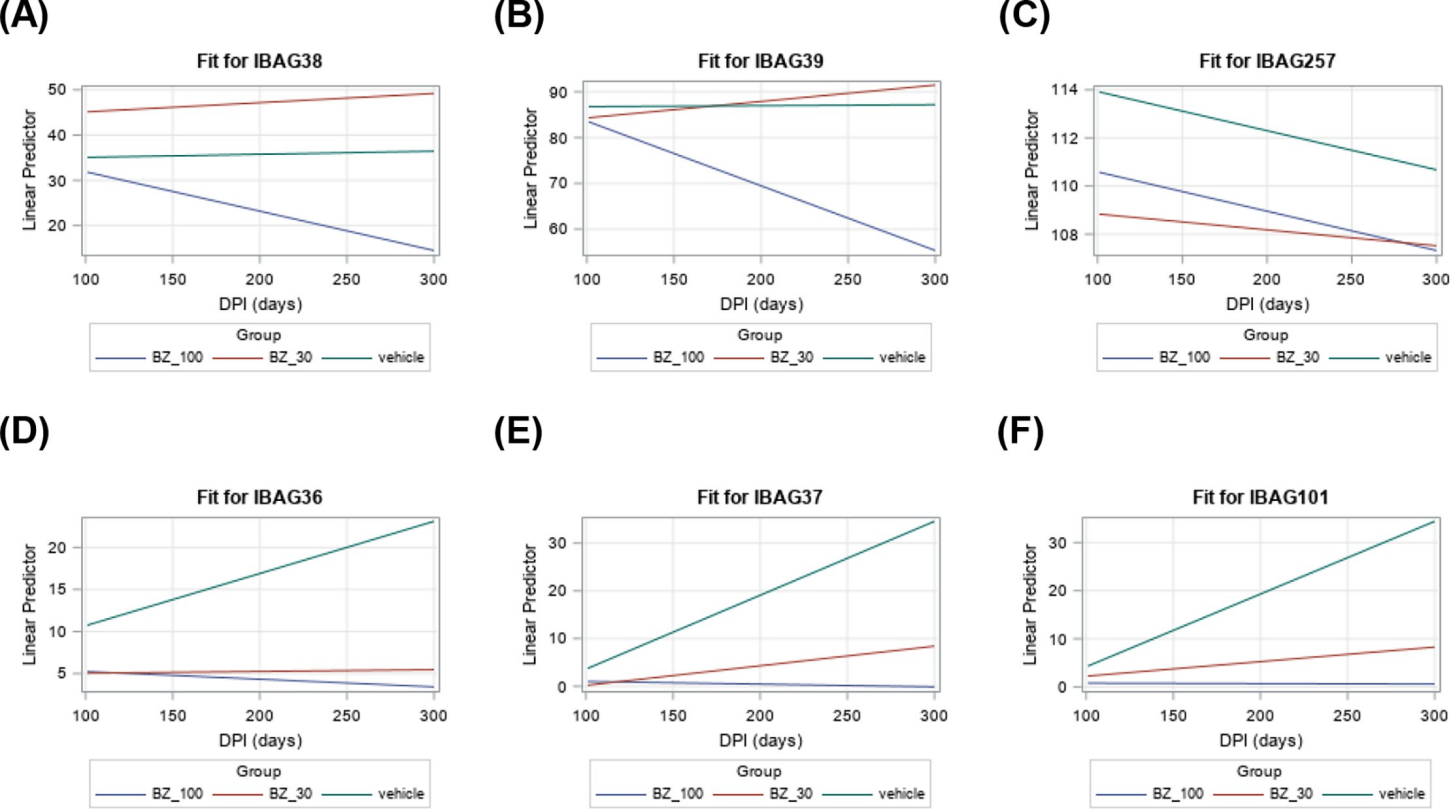
Average change of IBAG reactivity over time, based on the linear prediction model for mice treated with vehicle, and benznidazole at 30 and 100 mg kg^-1^. The fits correspond to the intercept and slopes in Table 1. The reactivity of (A) IBAG38 and (B) IBAG39 remains stable in mice treated with vehicle and benznidazole at 30 mg kg^-1^ and sharply decreases in 100 mg kg^-1^ benznidazole treated mice. (C) IBAG257 declines in reactivity in the three groups. The reactivities of (D) IBAG36, (E) IBAG37 and (F) IBAG101 increase only in vehicle-treated animals. The vertical axis is representative for the real intensity of the IBAGs.

At the initial screening time-point, 35 days post-infection, there was diversity in the antigen-specific response. Some antigens were detected by sera from all mice, whereas with other antigens, the response was universally weak or insignificant at this time-point (Fig 2A and 2B). Some antigens showed high signal intensities until the end of the monitoring period (300 days) (Fig 2C). These highly reactive antigens were IBAG39 (SAPA–shed acute phase antigen, a member of the trans-sialidase super-family expressed by infectious forms of the parasite) and IBAG257 (the B-cell superantigen Tc24, a flagellar calcium-binding protein), followed by IBAG38 (TcD) (Table 1 (see intercept), Figs 2C and 3). IBAG38 and IBAG39 antigen reactivities showed a significant decline over time in the 100 mg kg^-1^ benznidazole treated mice, but not in animals treated with the vehicle or 30 mg kg^-1^ benznidazole (Fig 3A and 3B). These antigens might be considered as sensitive to treatment. In contrast, IBAG257 reactivity showed a decline of only borderline significance in both the 100 mg kg^-1^ benznidazole and the vehicle-treated subgroups (Fig 3C). Therefore, we consider this antigen as non-sensitive to treatment, and highly reactive to the continued presence of antibodies. IBAG108 and IBAG131 were reactive, but no difference in longitudinal profile between subgroups could be observed.

For three other antigens (IBAG36, IBAG37 and IBAG101), significant reactivity only became apparent 135 days post-infection in the vehicle control group, followed by a slow but steady increase until 300 days post-infection (Fig 3D–3F). In contrast, in the mice treated with 100 mg kg^-1^ benznidazole, there was no detectable immune response against these antigens, while in mice that had been treated at 30 mg kg^-1^, a small increase was observed towards the end of the experimental period (Fig 3D–3F). These data indicate that IBAG36, IBAG37 and IBAG101 represent specific markers for late stage chronic infection and immune reactivity to these antigens can be reduced or eliminated by benznidazole treatment in a dose-dependent manner. Approximately 20% of mice recognised antigen IBAG112 by day 100 post-infection, but by day 300, this reactivity was on a downward trajectory. For the other antigens (IBAG35, IBAG95, IBAG97, IBAG99, IBAG112, IBAG20, IBAG134, and IBAG110), no significant reactivity was recorded at the different time points tested throughout infection (Table 1 and Fig 2C).

### Correlation between bioluminescence intensities and individual serology biomarkers

**Fig 4:**
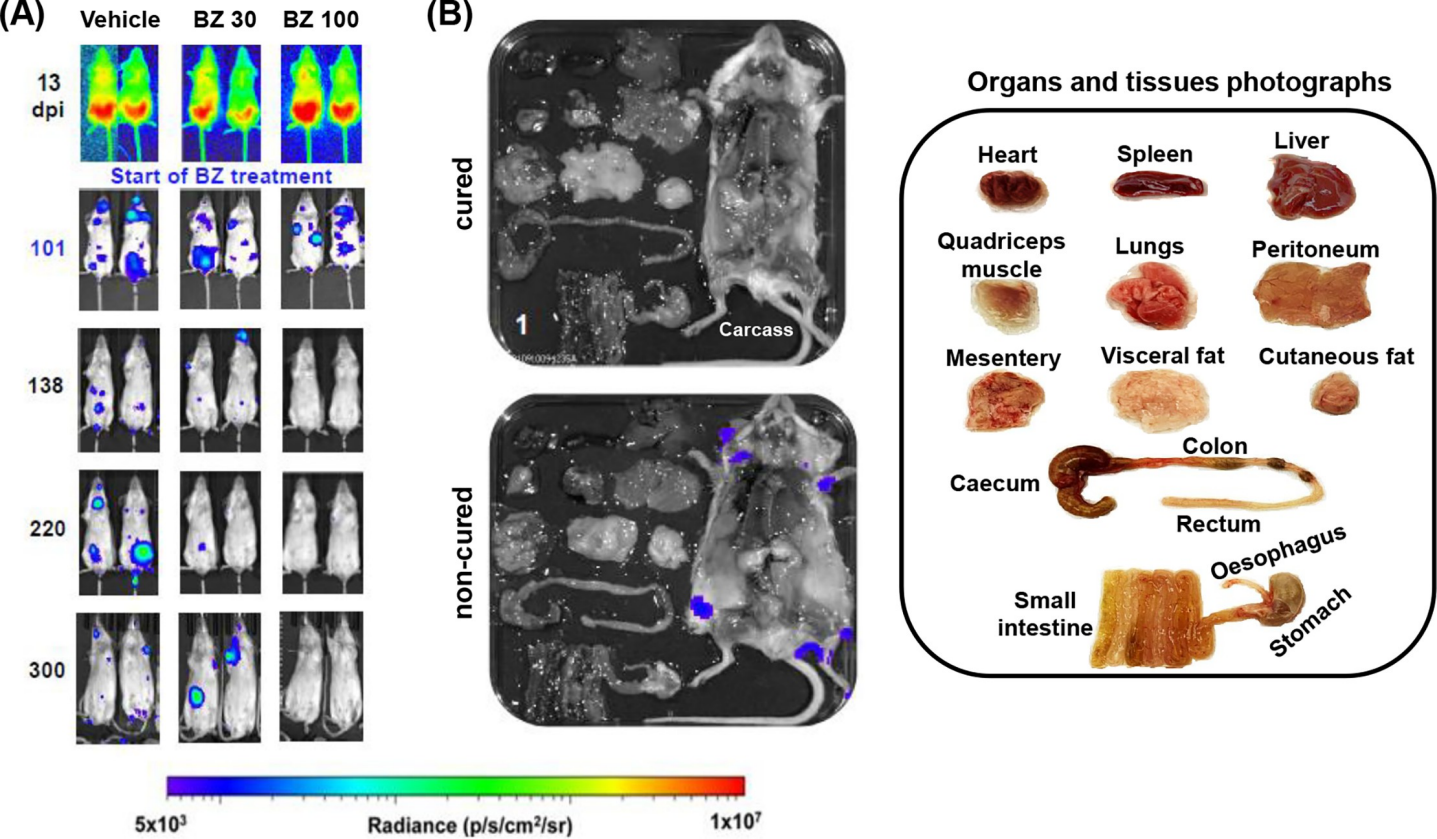
*In vivo* and *ex vivo* bioluminescence imaging of benznidazole-treated *Trypanosoma cruzi* infected mice. BALB/c mice were infected with bioluminescent *T*. *cruzi* (CL Brener strain) (Methods). 101 days post-infection (dpi), they were treated with benznidazole (BZ) at 30 or 100 mg kg^-1^, once daily for 5 days by the oral route (n = 15 per group). In parallel, a control cohort was administered with the HPMC vehicle (Methods). The mice were monitored regularly by bioluminescence imaging. (A) Representative ventral images of two mice per group (see also S1 Fig). (B) At the experimental end-point, mice were euthanized and organs and tissues were arrayed in a square petri dish, bathed in d-luciferin, and subjected to *ex vivo* imaging (Methods). The organs and tissues are organized as shown in the inset (right). See S2 Fig for other examples. All images use the same log10 scale heat-map with minimum and maximum radiance values as indicated. Mice that were bioluminescence negative at the experimental end-point by both *in vivo* and *ex vivo* imaging were designated as cured.

As bioluminescence imaging is an accepted reference method for monitoring parasite burden in mice, we next assessed the linear correlation coefficient for each reactive antigen and bioluminescence data after the initiation of benznidazole treatment. Overall, treatment resulted in a reduction in the bioluminescence-inferred parasite burden close to background levels (Figs 4 and S1). However, by 300 days post-infection, the experimental end-point, all mice treated at 30 mg kg^-1^ had relapsed (n = 15) (S1 and S3 Figs). In contrast, mice treated at 100 mg kg^-1^ were bioluminescence-negative by *in vivo* imaging at day 300 (Figs 4, S2 and S3). When these mice were further assessed by *ex vivo* imaging, small bioluminescent foci were detectable in the carcass of 4 animals, with the other 11 mice being bioluminescence-negative (Figs 4B and S2). This latter group was designated as cured. We did not use immunosuppression to promote relapse in this study, because of the long post-treatment period (almost 200 days), and because of concerns that it would perturb the immunoassay readout.

**Fig 5:**
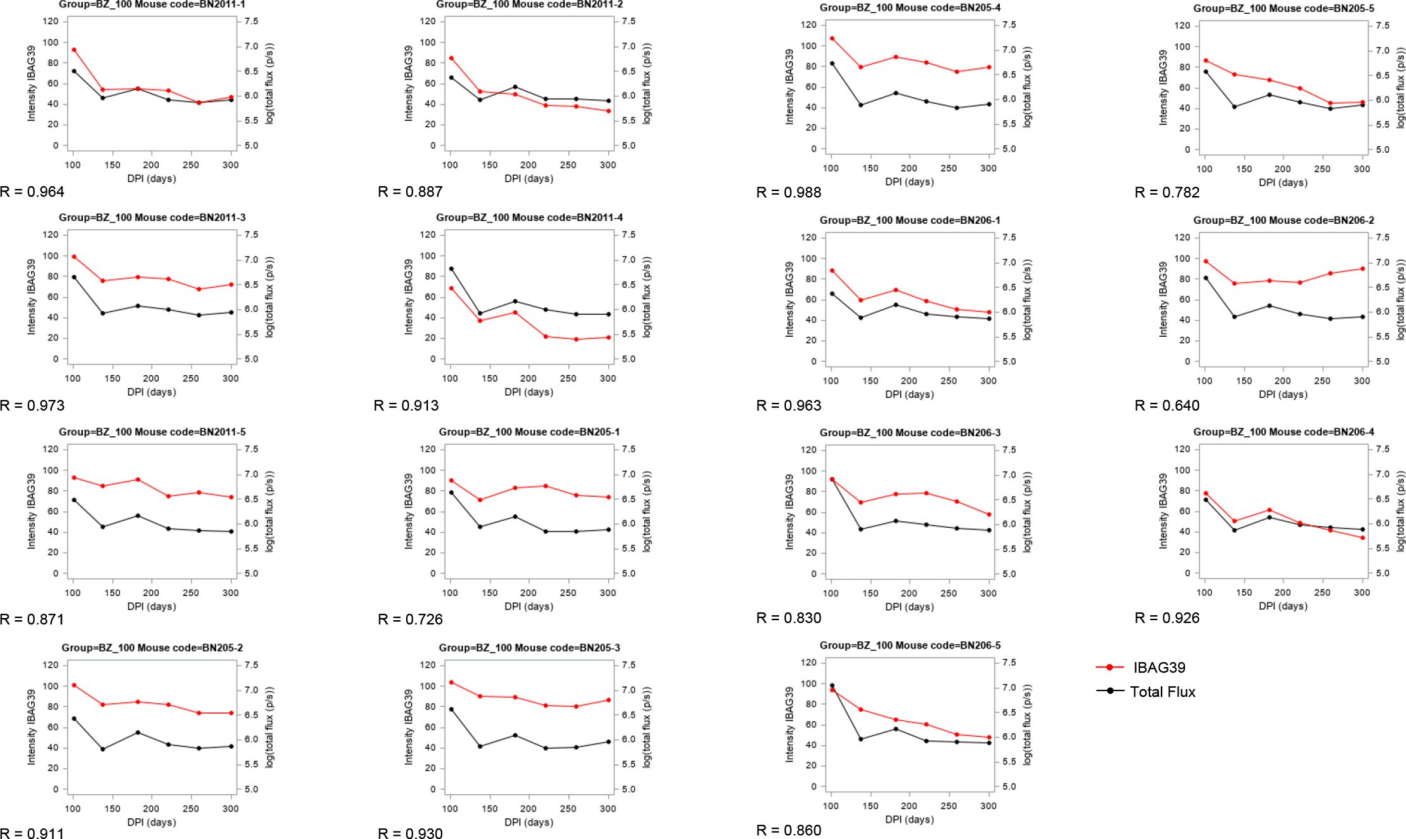
Bioluminescence total flux and IBAG39 intensity of reactivity for each of the 15 mice treated with 100 mg kg^-1^ benznidazole (BZ). The linear correlation coefficient R is given in each graph. The red curve is intensity of IBAG39 and the black curve is log (total flux (p/s)).

Table 2 shows the linear correlation coefficients for individual biomarkers and bioluminescence data from each mouse. Strikingly, the analysis revealed a very high linear correlation between antigen reactivities of IBAG39 and the log-transformed bioluminescence variable in mice treated with 100 mg kg^-1^ benznidazole, indicating that IBAG39 is a potential therapy monitoring biomarker (Fig 5).

**Table 2:** Linear correlation coefficients of each antigen for each infected mouse treated with 100 mg kg^-1^ benznidazole. Correlation coefficients were calculated, for log10 (total flux) and biomarkers, for each mouse separately (n = 6 observations post-treatment). Statistically significant correlation coefficients are marked in red. *Red*: linear correlation coefficients > 0.8 (p-value < 0.05).

Linear correlation coefficients																
Mouse ID	IBAG 35	IBAG 36	IBAG 37	IBAG 38	IBAG 39	IBAG 95	IBAG 97	IBAG 99	IBAG 101	IBAG 112	IBAG 20	IBAG 134	IBAG 110	IBAG 108	IBAG 131	IBAG 257
BN2011-1	0.347	0.587	0.854	0.811	0.964	0.884	0.188	0.748	0.931	0.946	0.784	-0.633	-0.110	0.860	0.932	0.481
BN2011-2	0.903	0.717	0.161	0.901	0.887	0.605	0.732	0.400	-0.070	0.844	0.512	-0.392	0.566	0.833	-0.016	0.485
BN2011-3	0.913	0.392	0.216	0.973	0.973	0.437	0.226	-0.038	-0.131	0.929	-0.248	-0.542	-0.241	-0.559	-0.585	0.073
BN2011-4	0.469	0.809	0.489	0.924	0.913	0.462	0.111	0.246	0.241	0.223	0.902	0.281	-0.292	0.429	0.148	0.100
BN2011-5	0.963	-0.487	0.939	0.953	0.871	0.863	-0.241	-0.185	0.970	0.367	-0.501	0.184	-0.340	-0.205	0.995	-0.140
BN205-1	0.895	0.408	0.980	0.767	0.726	0.808	0.030	-0.252	-0.087	0.944	-0.290	-0.068	0.346	0.153	0.852	0.219
BN205-2	0.844	0.827	0.831	0.853	0.911	0.690	-0.307	0.468	0.884	-0.198	‐0.863	‐0.845	-0.470	0.894	0.349	0.692
BN205-3	0.896	0.888	0.467	0.946	0.930	0.854	0.827	0.323	0.934	-0.308	-0.400	-0.191	-0.283	-0.211	0.457	0.861
BN205-4	0.712	0.978	0.394	0.976	0.988	0.431	0.543	0.811	0.822	-	-0.131	0.842	-0.427	-0.193	0.779	0.897
BN205-5	0.844	0.798	0.203	0.585	0.782	0.039	0.022	-0.332	0.893	0.136	0.650	0.915	-0.250	0.730	0.050	0.414
BN206-1	0.370	0.826	-0.430	0.807	0.963	0.699	0.856	-0.017	0.229	-0.799	0.039	0.353	0.293	-0.187	0.268	0.224
BN206-2	0.030	0.373	0.046	0.640	0.640	0.495	-0.040	0.627	-0.227	0.784	0.246	0.141	-0.001	-0.129	-0.521	-0.321
BN206-3	0.784	0.624	0.068	0.378	0.830	0.719	0.592	0.423	-0.090	-0.043	-0.386	0.678	-0.306	0.498	0.497	-0.034
BN206-4	0.572	0.947	0.113	0.182	0.926	0.401	0.060	0.472	0.273	-0.199	-0.552	0.178	-0.403	-0.458	0.003	0.138
BN206-5	0.038	0.556	0.132	0.842	0.860	-0.064	0.116	-0.248	-0.769	0.281	-0.355	-0.363	0.817	‐0.887	-0.394	0.588

## Discussion

This study addresses one of the key challenges in Chagas disease control, which is the lack of serological tools and reliable biomarkers to monitor parasitological cure. The current strategy, for investigational settings, is to use PCR-based methods for assessing Chagas disease drug efficacy. However, because of the highly focal and low-level nature of chronic infections [22, 27], long-term follow-up is required [16, 17], and the potential for “false-cure” outcomes remains high. This presents major problems in clinical trials, and in future, will complicate the roll-out of new therapies. The recently developed multiplex serology assay system has shown potential as a means of monitoring *T*. *cruzi* persistence and identifying biomarkers that are predictive of successful treatment [29–31]. To further explore the utility of this approach, we applied the multiplex methodology to a highly sensitive experimental infection model, based on bioluminescence, where the limit of detection by *ex vivo* imaging is less than 20 parasites [22]. The aim was to determine if procedures such as this could be incorporated into the drug development pipeline, as an additional approach to identifying drug candidates that eliminate an infection. This experimental system also has the advantage of a standardized infection timeline and a method for designating cure that is more reliable than PCR-based methodologies [34].

During the acute stage, *T*. *cruzi* infection induces a strong polyclonal B cell/antibody response which can protect against virulent infection [35]. In the chronic stage however, the role of the humoral immune system is less certain [36]. The difficulties in addressing this reflect the diversity and complexity of the antibody response, even within an in-bred mouse population infected by a single *T*. *cruzi* clone (Fig 2). As shown, responses to some parasite antigens are rapid and almost universal (IBAG38, IBAG39, IBAG108 and IBAG257), whereas with others (*eg*. IBAG36, IBAG37 and IBAG101), induction is slow, but progressive. One of the most reactive antigens identified in the multiplex assay, IBAG257, corresponds to Tc24, a flagellar-localised Ca^2+^ binding protein that is highly conserved among different *T*. *cruzi* strains and is expressed throughout the development cycle [37, 38]. It has been widely tested as a vaccine candidate [39–41], and its use in serodiagnosis has been reported [42, 43]. Here, we found that the reactivity to this antigen using the multiplex assay was rapid, high and ubiquitous (n = 45) (Fig 2), but did not display discriminatory power to identify treated mice. A similar rapid and robust response was observed to IBAG39 (SAPA [44, 45]) and IBAG38, with all 45 mice in the current study (Fig 2), and all 25 mice in the pilot experiment, generating reactive antibodies. However, it was possible to discriminate between treated and non-treated mice on the basis of the reactivity towards these antigens. In response to 100 mg kg^-1^ benznidazole treatment, there was a slow but steady decline in the intensity of IBAG38 and IBAG39 antibody reactivity post-dosing, compared to a continued increase in the non-drug treated mice (Fig 3A and 3B), a profile that was highly correlated (for IBAG39) with the bioluminescence flux (Fig 5). The response was intermediate in the case of mice treated at 30 mg kg^-1^.

Non-treated mice could also be discriminated by their response to IBAG36, IBAG37 and IBAG101. In the responding mice, the net intensity increased progressively from day 100 post-infection onwards, whereas there was no significant response in those mice that had been treated with 100 mg kg^-1^ (Fig 3D–3F). IBAG108 proved to be highly reactive, with a rapid and long-lasting response in >90% of the mice tested (Fig 2C), but showed no difference between treatment subgroups. Therefore, in the context of the multiplex system, IBAG38, IBAG39, IBAG108 and IBAG257 represent a highly sensitive and powerful combination for diagnosis of both acute and chronic *T*. *cruzi* infection, with IBAG39 having potential as a therapy-monitoring biomarker in mice. The multiplex system approach offers significant value for biomarker identification and validation due to its multi-parametric nature, with information collected simultaneously for several different antigens. Each antigen provides data on the development of reactivity and the probable antibody load in individual animals. The combination of independent parameters, enhances the analysis and provides a rapid, reliable and robust indication of signal decay linked to waning antibody levels. This approach, combined with recently developed prediction and dilution concepts based on mathematical models, is especially relevant for rapid evaluation of the dynamics of the antibody-mediated immune response [31, 46]. Treatment success is difficult to measure using the current criterion for parasitological cure because *T*. *cruzi*-specific antibodies persist for long periods after successful anti-parasitic treatment, and long-term follow-up is needed to monitor seroconversion.

Detailed validation of the Multi-Cruzi immunoassay and selected clinically proven biomarkers is important as a demonstration of translational value—its usefulness for *in vivo* basic research and diagnostic applications for patients. One limitation of the current study concerns the restriction to the BALB/c mouse infection model and the *T*. *cruzi* CL Brener strain. Another could be the reported immune modulation associated with benznidazole treatment [47]. It will therefore be worthwhile to verify whether reduction of parasite load is also associated with a decrease of the specific serological markers when using other *T*. *cruzi* lines, mouse strains, and therapeutic agents. Finally, it would be useful to extend our study to other laboratory animals, such as naturally infected non-human primates, which can serve as a model for evaluating the effect of drug candidates on infection with this parasite [48], and for informing strategies applicable to a clinical context.

In summary, the Multi-cruzi assay represents a highly promising research and discovery tool that can be used in combination with currently used diagnostic techniques (PCR and bioluminescence) to perform serological profiling studies and to monitor drug and possibly vaccine efficacy in preclinical models. Furthermore, its early adoption during the discovery process, and as a tool for clinical trial evaluation and regulatory approval, could help to drive acceptance of new treatments for Chagas disease.

## Supporting information

S1 Fig Longitudinal monitoring of *Trypanosoma cruzi*-infected mice by *in vivo* bioluminescence imaging. 45 BALB/c mice were infected with bioluminescent *T*. *cruzi* (strain CL Brener) (Methods). At day 101 post-infection (dpi) (indicated by red arrow), the mice were treated with vehicle, 30 or 100 mg kg^-1^ benznidazole (BZ), once daily by the oral route for 5 days (n = 15 per group). They were monitored by *in vivo* imaging until the experimental end-point (Methods). For each group of 15 mice, ventral and dorsal images were captured in sets of 5, numbered left to right (#1–5, 6–10 and 11–15). All images use the same log10 scale heat-map with minimum and maximum radiance values as indicated.
(PPTX)

S2 Fig *Ex vivo* imaging of benznidazole-treated mice at the experimental end-point. At 301 days post infection, mice that had been treated with 100 mg kg^-1^ benznidazole for 5 days (S1 Fig) were euthanized and subjected to *ex vivo* imaging (Methods). Bioluminescent foci (examples highlighted by white arrows) were detected in 4 mice, which were designated as non-cured. The organs and tissues are organized as shown in Fig 4.
(PPTX)

S3 Fig Total whole body bioluminescence of mice at the experimental end-point. Mice (n = 15 per group) were imaged 300 days post-infection to establish whole body bioluminescence (sum of ventral and dorsal images) (Methods). (A) Control cohort administered with HPMC vehicle; (B) and (C) Cohorts treated with benznidazole (BZ) at 30 and 100 mg kg ^-1^, respectively (Methods). See S1 Fig, to identify *in vivo* images of individual mice (numbering system described in the legend). In the 100 mg kg^-1^ cohort, the (+) symbol identifies mice shown to be non-cured by *ex vivo* imaging (S2 Fig). Dashed lines represent average background levels for naïve mice (n = 5).
(PPTX)
