# Supplementary material for: Comparing in vivo bioluminescence imaging and the Multi-Cruzi immunoassay platform to develop improved Chagas disease diagnostic procedures and biomarkers for monitoring parasitological cure
Source: PLoS Negl Trop Dis. 2022 Oct 3;16(10):e0010827. doi: 10.1371/journal.pntd.0010827 (PMC9560623; doi:10.1371/journal.pntd.0010827)
Supplement: S1 Fig — 45 BALB/c mice were infected with bioluminescent T. cruzi (strain CL Brener) (Methods). At day 101 post-infection (dpi) (indicated by red arrow), the mice were treated with vehicle, 30 or 100 mg kg-1 benznidazole (BZ), once daily by the oral route for 5 days (n = 15 per group). They were monitored by in vivo imaging until the experimental end-point (Methods). For each group of 15 mice, ventral and dorsal images were captured in sets of 5, numbered left to right (#1–5, 6–10 and 11–15). All images use the same log10 scale heat-map with minimum and maximum radiance values as indicated. (PPTX) [file pntd.0010827.s001.pptx]

## Slide 1
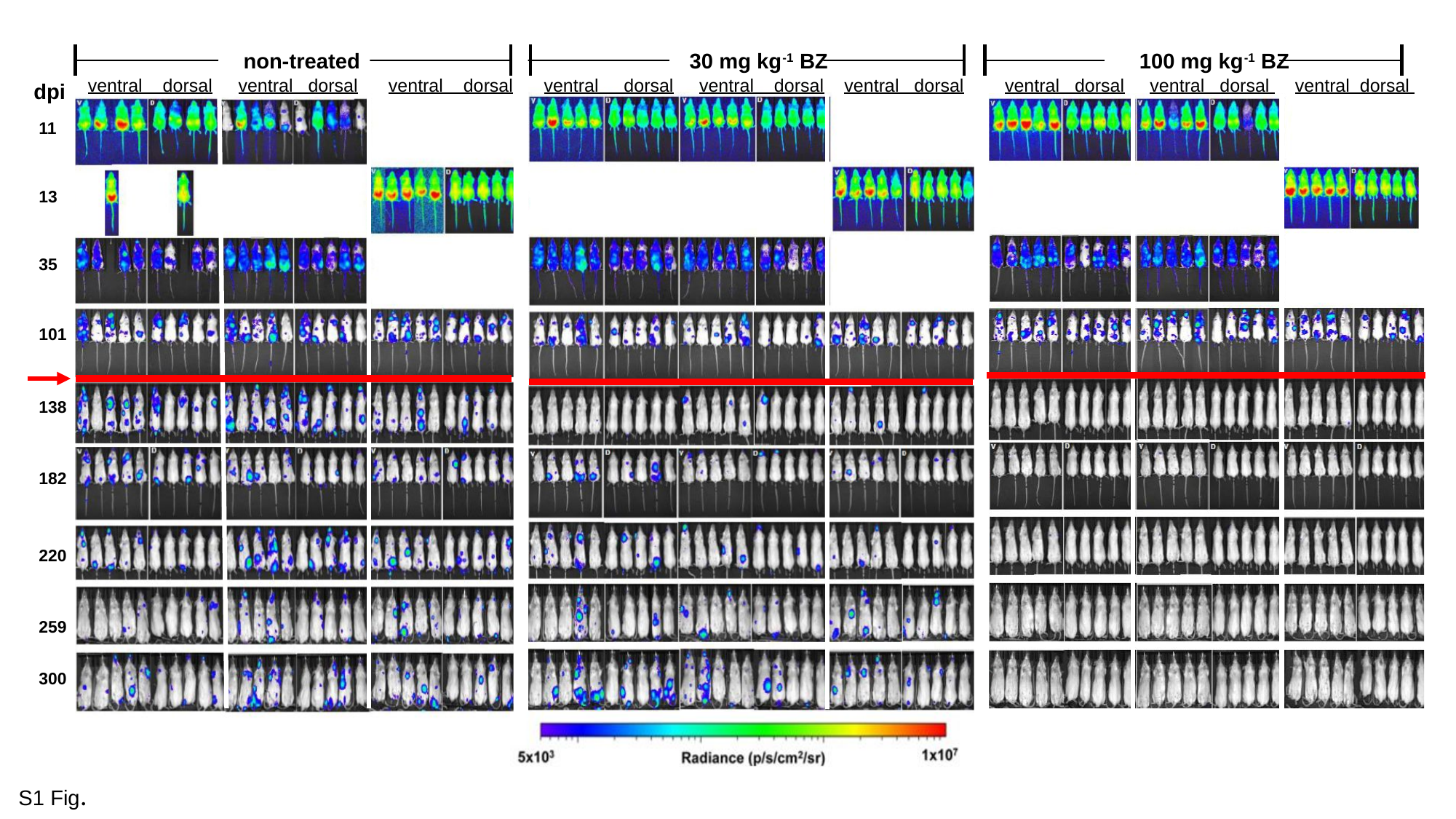

non-treated 30 mg kg-1 BZ 100 mg kg-1 BZ
ventral dorsal ventral dorsal ventral dorsal ventral dorsal ventral dorsal ventral dorsal ventral dorsal ventral dorsal ventral dorsal
dpi
11
13
35
101
138
182
220
259
300
S1 Fig.
