# Supplementary figures and images for: Comparing in vivo bioluminescence imaging and the Multi-Cruzi immunoassay platform to develop improved Chagas disease diagnostic procedures and biomarkers for monitoring parasitological cure
Source: PLoS Negl Trop Dis. 2022 Oct 3;16(10):e0010827. doi: 10.1371/journal.pntd.0010827 (PMC9560623; doi:10.1371/journal.pntd.0010827)

## Slide 1
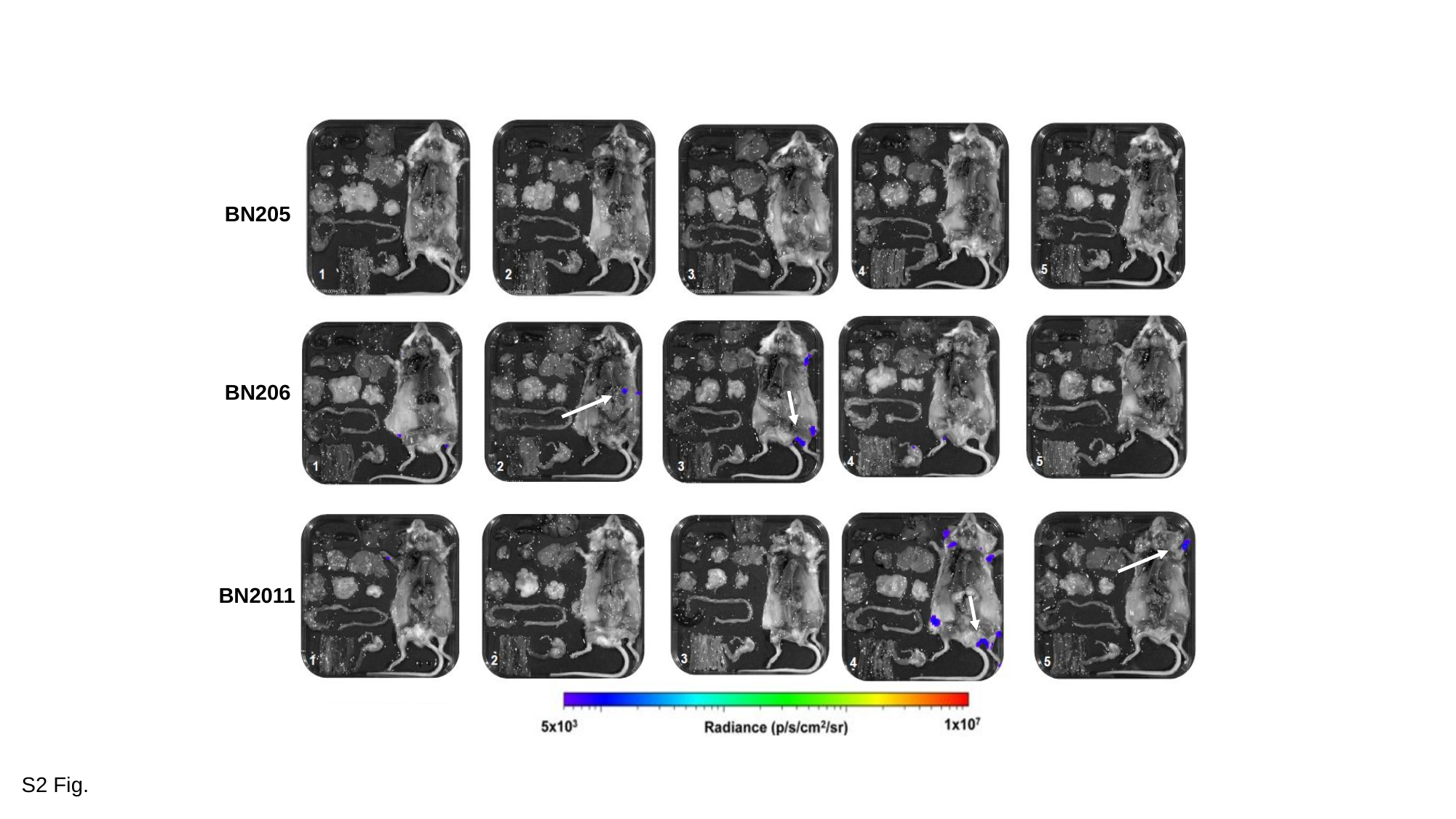

BN205
 BN206
BN2011
S2 Fig.

Supplement: S2 Fig — At 301 days post infection, mice that had been treated with 100 mg kg-1 benznidazole for 5 days (S1 Fig) were euthanized and subjected to ex vivo imaging (Methods). Bioluminescent foci (examples highlighted by white arrows) were detected in 4 mice, which were designated as non-cured. The organs and tissues are organized as shown in Fig 4. (PPTX) [file pntd.0010827.s002.pptx]

## Slide 1
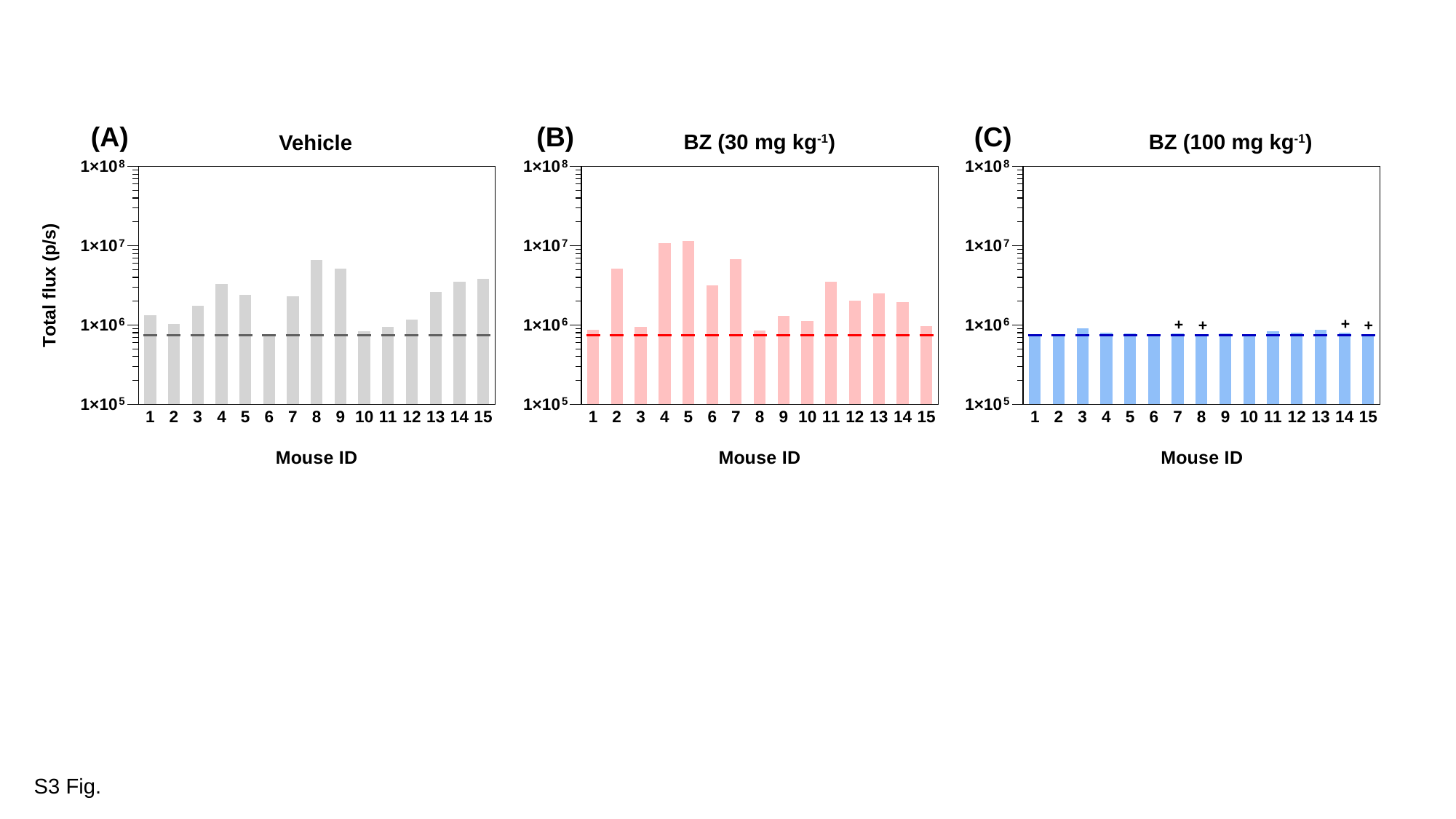

(A)
(B)
(C)
BZ (100 mg kg-1)
BZ (30 mg kg-1)
Vehicle
S3 Fig.

Supplement: S3 Fig — Mice (n = 15 per group) were imaged 300 days post-infection to establish whole body bioluminescence (sum of ventral and dorsal images) (Methods). (A) Control cohort administered with HPMC vehicle; (B) and (C) Cohorts treated with benznidazole (BZ) at 30 and 100 mg kg -1, respectively (Methods). See S1 Fig, to identify in vivo images of individual mice (numbering system described in the legend). In the 100 mg kg-1 cohort, the (+) symbol identifies mice shown to be non-cured by ex vivo imaging (S2 Fig). Dashed lines represent average background levels for naïve mice (n = 5). (PPTX) [file pntd.0010827.s003.pptx]
